# Estimation of microbial protein synthesis in the rumen of growing lambs based on the purine derivative excretions and the dietary forage-to-concentrate ratio

**DOI:** 10.5455/javar.2023.j691

**Published:** 2023-09-24

**Authors:** Zahra Mahboobi, Naser Karimi, Abbas Jahanbakhshi

**Affiliations:** Department of Animal Science, Varamin-Pishva Branch, Islamic Azad University, Varamin, Iran

**Keywords:** Growing lamb, microbial protein synthesis, purine derivatives

## Abstract

**Objective:**

Estimating microbial protein synthesis (MPS) in the rumen of growing lambs based on the urinary excretion of purine derivatives (PDs) and forage to concentrate (F/C) ratio.

**Materials and Methods:**

36 similar-growing male lambs (weight 32.53 ± 1.90 kg; age 93 ± 6.63 days) were used in a completely randomized design with four groups: a) the 20–80 F/C ratio (dry hay 10% + wheat straw 10%), b) the 20–80 F/C ratio (dry hay 0% + wheat straw 20%), c) the 10–90 F/C ratio (dry hay 5% + wheat straw 5%), d) and the 10–90 F/C ratio (dry hay 0% + wheat straw 10%) with nine replicates.

**Results:**

Total PD and rumen MPS synthesized increased (10.98 *vs.* 13.25 mmol/day and 59.45 *vs*. 71.80 gm/day) in group d compared to group a. Dry organic matter intake (0.869 kg/day), fermentable dry organic matter (0.563 kg/day), and microbial nitrogen (N) yield (11.48 gm/day) of group d were at the maximum, but in terms of gN/kg dry organic matter (22.37 gm/kg), the mean of group c was higher than others.

**Conclusion:**

Increasing the level of food concentrate and the gradual removal of alfalfa from the diet increased the excretion of PD and MPS in the rumen. It was also found that urinary PD monitoring is an accurate indicator for the estimation of MPS.

## Introduction

Microbial protein synthesis (MPS) is of special importance in ruminants. Because it supplies an important part of the protein and essential amino acids needed by ruminants [1,2]. From an economic point of view, it is important to maximize MPS in the rumen from low-quality protein or nonprotein nitrogen (NPN). However, in mixed farming systems with poor digestibility, low protein, and high fiber content diets, livestock production improvement requires efforts to maximize feed efficiency by using appropriate concentrates to meet acceptable results for rumen microbes. Therefore, adopting efficiency and providing favorable conditions for the growth of rumen microbial life is necessary to maximize the use of nutrients [3,4]. Two main protein sources provide the protein required by ruminants. The first is the decomposition of feed proteins in the rumen and conversion into a microbial protein named rumially degradable protein (RDP), and the second is the form of transition protein, or rumially un-degradable protein, which is transferred from the rumen to the small intestine without any changes [1,5,6]. The amount of protein synthesized by rumen microorganisms is generally related to the provision of fermentable energy, usable NPN, essential vitamins, minerals, and micronutrients [5,7].

Previous studies have mainly focused on diet digestibility, the pattern of rumen fermentation, and/or the kinetics of digesta passage, but rumen MPS and nitrogen balance have received much less attention despite their influence on animal health and production. Nitrogen excretion represents an important source of environmental contamination in every farming system. Excretion of nitrogen in feces and urine depends mainly on the nitrogen content of the diet and its digestibility, but it may also vary with animal species [8]. Many studies have also estimated the outflow of microbial protein from the rumen using techniques such as ineffective markers or fistulated animals. However, these techniques are difficult to perform and have not led to robust models for predicting MPS as part of ration models [4,6,7]. Typically, estimation of microbial yield based on the methods mentioned with a few rumen or duodenum-cannulated animals is a time-consuming procedure with a high standard error of estimates [9–11].

Rumen microbes are responsible for breaking down the majority of dietary purine bases. Therefore, most purines absorbed, broken down in the small intestine, and excreted through the urine are of microbial origin [12–14]. Some previous studies reported the effect of supplements, forage, and/or feed restrictions on the percentage of urinary purine base derivatives. However, the effect of different F/C ratios on the relationship between urinary purine derivative excretion and the intestinal microbial protein has been less investigated [15–17]. According to the National Research Council (NRC) [5], in forage-concentrate mixed diets, the mean efficiency of MPS is 17.6 gm per 100 gm of digestible organic matter, and the efficiency of MPS for diets with a high amount of concentrate is 13.2 gm per 100 gm of digestible organic matter.

If there is a shortage of available forage, it is necessary to consume more dietary concentrate. Of course, in this situation, the necessary points in feeding the concentrate and its ratio with forage must be carefully observed. Also, fodder, due to its fibrous nature and insufficient amount of energy and protein for the growth of fattening lambs, cannot fully provide the nutrients needed by these animals [18,19]. Therefore, it is necessary to supply a lack of energy and protein by concentrating. Chen and Gomes [9] stated that the efficiency of MPS increases due to an increase in the passage rate of digestible rumen materials. The efficiency of MPS when consumed as high-quality forage by animals is often recorded to be high (30–45 gm of microbial nitrogen per kg of digestible organic matter), while the efficiency of MPS is much lower than 20% when low-quality forage is consumed by the animals [20], as reported previously [21,22].

The outflow of MN to the duodenum can be estimated by the urinary excretion of PD and response models developed in sheep [9–11,23–26] and goats [6,12,13,26]. However, studies with sheep and goats fed different F/C ratios are scarce, with contrasting results 17. Our hypothesis focused on easier and noninvasive techniques for the estimation of MPS, as these methods have the potential to develop new diagnostic tests to commercialize research. Urinary PD assessment is a practical method to estimate rumen MN synthesis after changes in diet composition or F/C ratio [10,12,13]. According to our hypothesis, the reduction of the F/C ratio in the diet of Afshari-growing male lambs increases the intake of nutrients and increases the output of microbial protein to the duodenum, which in turn increases the excretion of PD in the urine. This reduction can prevent the negative effects of consuming low-quality fodder. Therefore, the purpose of this experiment was to investigate the effect of changing diet composition and F/C ratio on MPS using the method of evaluating the excretion of PDs in urine.

## Materials and Methods 

### Ethical approval 

All methods used in this study on animals were approved by the animal care and use committee of Islamic Azad University. Lambs were maintained according to the NRC guidelines for the care and use of laboratory animals (reference number: IAUEC 2021/1400-265-33-08).

### Experimental animals

This experiment was conducted from June to August 2021, using 36 Afshari-growing male lambs with an average weight of 32.53 ± 1.9 kg and an average age of 93 ± 6.63 days in a private sheep breeding unit in Varamin, Tehran, Iran.

### Management and diets

After weighing, the growing lambs were divided into four experimental groups, and each ration was randomly assigned to one of the groups based on a completely randomized statistical design (CRD). The experimental diets were as follows: a) 1. 80% concentrate and 20% forage (10% wheat straw and 10% dry alfalfa); b) 2. 80% concentrate and 20% forage (20% wheat straw and 0% dry alfalfa); c) 3. 90% concentrate and 10% forage (5% wheat straw and 5% dry alfalfa); and d) 4. 90% concentrate and 10% forage (10% wheat straw and 0% dry alfalfa). Energy, protein, calcium, phosphorus, and other nutrients needed by small ruminants were adjusted according to the nutrient requirements of the NRC [5] tables. To adjust the ration, first, the amount of dry matter (DM), crude protein (CP), and ash was estimated by the laboratory methods of Association of Official Analytical Chemists [23], and DM digestibility was calculated according to Agricultural Research Council (ARC) [24]. Ingredients and the chemical composition of experimental diets are shown in Table 1.

Diet components were weighed and then mixed into the total mixed ration. The chemical composition of diets was calculated and estimated using the chemical composition of food based on the NRC [5]. The lambs were fed *ad libitum* three times a day at 6-h intervals. The amount of feed given and the residues were weighed daily to determine the daily feed intake. The fresh and clean water was constantly available to the animals. The experimental period was 90 days, and the lambs were weighed once every 2 weeks.

**Table 1.** Feed ingredients of experimental diets.

		Diets^A^		
	D1	D2	D3	D4
Ingredients (gm/kg)				
Alfalfa hay	100	0	50.00	0
Wheat straw	100	200	50.00	100
Ground barley	304	304	360	360
Ground corn	304	296	351	351
Soybean meal-44% CP	72.00	72.00	76.50	81.00
Canola meal-35% CP	20.00	16.00	18.00	16.00
Fat powder^B^	28.00	28.00	27.00	27.00
Urea granule-46% N	13.60	17.60	13.50	13.50
Bentonite^C^	10.40	16.80	9.00	10.00
Calcium carbonate	18.40	18.40	22.50	18
Sodium bicarbonate	10.40	16.80	9.00	10.00
Edible salt	4.80	4.80	4.50	4.50
Vitamin and mineral permix^D^	14.40	9.60	9.00	9.00
Total	1,000	1,000	1,000	1,000
Chemical composition (% of DM unless otherwise noted)				
Dry matter	91.22	90.66	90.28	92.91
CP	18.41	18.4	18.49	18.14
Ash	10.93	9.98	7.82	7.95
NDFE	81.94	61.75	68.33	58.60
ADFF	13.45	12.62	7.30	9.20
Calcium	1.90	2.20	2.50	2.80
Posphorus	0.50	0.50	0.50	0.50
Metabolizable energy (Mcal/kg DM)	2.93	2.90	2.96	2.98

^A^Diets were included: Diet 1. Concentrate 80% + alfalfa hay 10% + wheat straw 10%; Diet 2. Concentrate 80% + alfalfa hay 0% + wheat straw 20%; Diet 3. Concentrate 90% + alfalfa hay 5% + wheat straw 5%; Diet 4. Concentrate 90% + alfalfa hay 0% + wheat straw 10%.

^B^Calcium salt of fatty acids (fat 84%, Ca 11%, moisture 5%) manufactured by Kimiya Roshd Chemical Industries, an Iranian chemical Co., Yellow granules with purity 88%, digestibility 90%, NEL 7.5 Mcal/kg DM.

^C^Bentonite manufactured by the Iranian Chemical Industry (general formula: nAl_2_O_3_.mSiO_2_.xH_2_O), from the aqueous aluminosilicate category with DM = 90%.

^D^Contained per kg of supplement: 1,300,000 IU of vitamin A, 360,000 IU of vitamin D, 1,200 IU of vitamin E, 16 gm of Zn, 10 gm of Mn, 0.8 gm of Fe, 0.12 gm of Co, 0.15 gm of I, and 0.08 gm of Se.

^E^NDF: Neutral detergent fiber.

^F^ADF: Acid detergent fiber.

### Urine collection method

All animals from each treatment (9 lambs) were selected for urine sampling (36 lambs in total). On the 45th day, urine samples were taken from lambs at a specified time. Urine was mixed in equal proportion with 10% sulfuric acid to keep the pH low, preserve purine base derivatives, and prevent the growth of microbes and fungi, and poured into special sampling containers with a capacity of about 50 ml. After recording the number of lambs and the date of sampling on the containers, the samples were kept at a temperature of 20°C until the experiment according to the procedures described in references [9–11,26].

### Estimation of PDs and MPS

The amount of absorbed purines was calculated by inserting the total urinary excreted PD into the equations proposed by Chen and Gomes [9] with minor modifications by other researchers [7,26] provided for sheep (Eq. 1).

TPD (mmol/d) = 0.84*X *(0.15 × MW × AF) (1)

In the above equation:

TPD = total purine derivatives excretion; MW = metabolic weight (body weight^0.75^); AF = adjusting factor (e^-0.25X^); X = daily purine absorption or PA (mmol/day) = microbial nitrogen yield or MN (gN/d) ÷ 0.727; MN also calculated as: 32 gm/kg dry organic matter (DOM) digestibility (DOMR) [24]; DOMR = feed intake (kg) × %DM content × %OM content × %OM digestibility × 0.60].

The basis of this method is to measure the excretion of PDs from urine. To eliminate daily changes, urine collection was done for five consecutive days on the tested animals. Urine uric acid was measured with a Pars Azmoun kit (1400031), urinary allantoin was measured according to the method described in the related reports [9,25], and xanthine and hypoxanthine were measured by enzymatic method using an automatic analyzer. In sheep, allantoin, uric acid, xanthine, and hypoxanthine are PDs. Therefore, to estimate the total amount of PDs, the excretion of each derivative was calculated based on millimoles per day. Finally, two equations based on previous studies, taking into account subsequent minor changes [9,25], were used to estimate the MN and microbial protein synthesized in the rumen (Eqs. 2 and 3).


(2) $$\mathrm{Microbial}\;N\;(\mathrm{gm}/\mathrm{day})=\frac{\left(\frac{[\mathrm{PD}\;\mathrm{production}–(0.385\times \mathrm{MW}\mathrm{)]}}{0.84}\right)\times 70}{1000\;\times \;0.83\;\times \;0.116}$$

Microbial CP (gm/day) = Microbial N (gm/day) × 6.25 (3)

In the above equations:

Microbial N = total microbial nitrogen synthesis; Microbial CP = total microbial crude protein production; PD production = total purine derivatives; MW = metabolic weight (body weight 0.75); 0.385 × BW^0.75^ = the net endogenous share of PDs; 0.84 = fixed number that is used to recovery of absorbed purines in urine; 70 = amount of purine nitrogen.

6.25 = nitrogen to protein conversion factor (a constant number used to convert the amount of nitrogen to CP equivalent); 1,000 = a constant number used to convert kilograms to gm; 0.83 = the degree of microbial protein digestibility; and 0.116 = ratio of purine nitrogen to total nitrogen in rumen microbes.

### Statistical analysis

All experimental data were analyzed using CRD statistical design with SAS 9.2 software [27]. Traits data were analyzed by one-way analysis of variance and using the MIXED method (Proc Mixed) with repetitive observations in time. The Shapiro–Wilk test was used to ensure the normal distribution of the data. The mean difference of the treatments was compared at the *p *< 0.05 level using Duncan's multiple range test.

## Results 

### Weight gain, feed intake, and growth traits

Table 2 shows the effect of different diets on the growth characteristics and feed intake of growing lambs. Based on the results, the effect of different experimental diets on the performance of growing lambs at 90 days was significant (*p* < 0.05). As the results show, following the increase in the proportion of concentrate in the diet, the daily feed intake in diet 4 has increased by 6.39% compared to diet 2.

### Organic matter digestibility, PDs, and MPS

In Table 3, the effects of different diets on PD excretion, DOM intake, DOMR, and MN yield are presented. The values of the traits are presented as the mean of five measurements with a SE.

Table 4 shows the effect of different diets on urinary excretion of PDs, daily MN production, and daily MPS. According to the results, the amount of allantoin, uric acid, xanthine, and hypoxanthine excretion, total PDs, absorbed microbial purine bases, MN, and synthesized microbial protein in growing lambs increased significantly following the increase in concentrate consumption (*p *< 0.01).

## Discussion

As the results of Table 2 show, different experimental diets had a significant effect on the growth performance traits of growing lambs at the age of 90 days. An increased dietary share of concentrate caused an increase in the daily feed intake of growing lambs. This was true for both diets with high concentrate levels (diets 2 and 4 with 1.72 and 1.83 kg/day, respectively) compared to diets with a low level of concentrate (diets 1 and 3 with 1.48 and 1.64 kg/day, respectively). This means that by increasing the amount of dietary concentrate, both daily feed intake and daily weight gain will increase [28,29]. Therefore, the better growth of lambs can be related to the increase in energy and protein in the experimental diets. It seems that in this experiment, growing lambs fed with diet 4 increased their feed consumption to reach physical satiety following the reduction of forage in the diet, and as a result, they gained a higher daily weight gain. Also, increased feed consumption following the increase in concentrate seems logical because the concentrate is more palatable and can be consumed faster by the animal compared to forage. The retention time of concentrate in the rumen is lower than that of fodder, so the effect of fullness that occurs after consuming fodder limits feed consumption [6,20,30]. An increased ruminal passage rate of digesta is another reason that affects intestinal digestion and finally increases feed intake [20,31,32]. The relative low feed intake of diet 1 can be related to factors such as the woodiness, texture, and physical nature of the feed, the low digestibility of the diet, the decrease in the food passage rate, and the increased time of feed retention in the rumen [6,19]. The volume of the rumen is one of the factors that limit the optional intake of DM. There are sensitive receptors in the rumen's wall that are stimulated against the stretching and expansion, thus reducing the movement and feed fermentation in the rumen, which will eventually lead to decreased feed consumption [20,29,31,32]. Increasing DM intake to increase the ratio of concentrate in the diet has often been confirmed in many experiments [16,17]. When the concentrate level of a dairy cow's diet is increased, a substitution effect is often achieved with large changes in the F/C ratio [15]. Also, it has been shown that when consuming relatively low-quality silage, the use of concentrate in higher than normal amounts resulted in increased feed intake due to the inclusion of a high level of dietary concentrate [33].

**Table 2.** The effect of experimental diets on daily feed intake and daily weight gain (gm/day) in 90-day-old growing lambs.

Items	Diets^A^				SEM	*p*-value
	D1	D2	D3	D4		
ILW^B^ (kg)	32.57	32.67	33.17	31.75	1.570	NS
FLW^C^ (kg)	51.20^b^	57.95^ab^	54.90^ab^	58.90^a^	1.730	0.003
DG^D^ (kg/day)	0.20^c^	0.28^a^	0.24^b^	0.30^a^	0.007	0.012
DFI^E^ (kg/day)	1.48^c^	1.72^ab^	1.64^b^	1.83^a^	0.051	0.035
FCR^F^	7.38^a^	6.15^c^	6.82^b^	6.10^c^	0.204	0.031

^abc^The different superscript letters on the top right of the values in the table indicate a significant difference of means at *p* < 0.05.

^A^Diets were included: D1. Concentrate 80% + alfalfa hay 10% + wheat straw 10%; D2. Concentrate 80% + alfalfa hay 0% + wheat straw 20%; D3. Concentrate 90% + alfalfa hay 5% + wheat straw 5%; D4. Concentrate 90% + alfalfa hay 0% + wheat straw 10%.

^B^Initial live weight.

^C^Final live weight.

^D^Daily gain.

^E^Daily feed intake.

^F^Feed conversion rate.

**Table 3.** The effect of different diets on PD excretion, DM intake, and MN yield.

Diets^A^	Mean body weight (kg)	DOMI^B^ (kg/day)	DOMR^C^ (kg/day)	PD excretion^D^ (mmol/day)	Microbial N yield	
gN/d^E^	gN/kg DOMR
Diet 1	41.88 ± 3.25	0.703 ± 0.07	0.457 ± 0.05	10.98 ± 1.25	9.51 ± 0.99	20.81 ± 2.29
Diet 2	45.31 ± 3.33	0.817 ± 0.09	0.531 ± 0.06	12.41 ± 1.41	10.75 ± 0.98	20.24 ± 2.23
Diet 3	44.04 ± 3.28	0.779 ± 0.08	0.506 ± 0.05	13.06 ± 1.52	11.32 ± 1.10	22.37 ± 2.41
Diet 4	45.32 ± 3.41	0.869 ± 0.09	0.565 ± 0.07	13.25 ± 1.55	11.48 ± 1.21	20.32 ± 2.22

All values are presented as mean ± SE of five measurements.

^A^Diets were included: D1. Concentrate 80% + alfalfa hay 10% + wheat straw 10%; D2. Concentrate 80% + alfalfa hay 0% + wheat straw 20%; D3. Concentrate 90% + alfalfa hay 5% + wheat straw 5%; D4. Concentrate 90% + alfalfa hay 0% + wheat straw 10%.

^B^DOMI [digestible organic matter intake = Feed intake (kg) × DM content (0.90) × OM content (0.88) × OM digestibility (0.60)].

^C^DOMR (organic matter apparently fermented in the rumen) was taken as 0.65 DOMI [24].

^D^PD excretion is the sum of uric acid, allantoin, xanthine, and hypoxanthine, based on Equation (1).

^E^Calculation were made based on Equation (2).

Our findings are in accordance with the results of some existing studies [6,8,14,17,19,28]. The best performance and carcass quality of fattening Merino lambs at the level of concentrate to straw as a fodder source with a ratio of 90:10 were reported [28]. The effect of different F/C ratios on growth performance, digestibility of nutrients, and ruminal parameters has been well investigated through relevant reports. The current findings show that increased dietary concentrate levels in diets led to increased final and daily weight gain of lambs, as well as a decreased feed conversion ratio and length of the fattening period [6,8,19,28]. This may be due to the lack of physical satiety of the animal due to not filling the rumen due to the consumption of concentrate compared to forage [31].

**Table 4.** The effect of different diets on the amount of allantoin (mmol/day), uric acid (mmol/day), xanthine and hypoxanthine (gm/day), total PDs (mmol/day), absorbed microbial purine (mmol/day), MN (gm/day) and microbial protein (gm/day) in growing lambs.

Items	Diets^A^				SEM	^p^-value
	D1	D2	D3	D4		
Allantoin (mmol/day)	7.68^c^	8.91^b^	9.01^ab^	9.25^a^	0.230	0.011
UA^B^ (mmol/day)	2.29^c^	2.35^b^	2.45^ab^	2.55^a^	0.001	0.000
X-H^C^ (gm/day)	1.01^c^	1.15^b^	1.60^ab^	1.45^a^	0.024	0.012
TPD^D^ (mmol/day)	10.98^c^	12.41^b^	13.06^ab^	13.25^a^	0.390	0.020
AMP^E^ (mmol/day)	13.08^c^	14.78^b^	15.55^ab^	15.78^a^	0.460	0.033
MN^F^ (gm/day)	9.51^a^	10.75^b^	11.31^ab^	11.48^a^	0.350	0.011
MPS-45^G^ (gm/day)	59.45^c^	67.22^b^	70.70^ab^	71.80^a^	1.090	0.000

^abc^The different superscript letters on the top right of the values in the table indicate a significant difference of means at *p* < 0.05.

^A^Diets were included: D1. Concentrate 80% + alfalfa hay 10% + wheat straw 10%; D2. Concentrate 80% + alfalfa hay 0% + wheat straw 20%; D3. Concentrate 90% + alfalfa hay 5% + wheat straw 5%; D4. Concentrate 90% + alfalfa hay 0% + wheat straw 10%.

^B^Uric acid.

^C^Xanthine and hypoxanthine.

^D^Total purine derivatives.

^E^Absorbed microbial purines.

^F^Microbial nitrogen.

^G^Microbial protein synthesis at age 45 days.

According to the report of Desnoyers et al. [30], the DM consumption of dairy goats increased from 2.69 to 2.88 kg/day following the increased dietary percentage of concentrate from 30% to 60%. Also, the effect of different F/C ratios (70:30, 60:40, 50:50, and 40:60) on the performance of dairy cows fed corn silage was investigated. Our findings are consistent with some results that indicate a significant increase in feed consumption and daily weight gain following an increase in dietary concentrate [6,17,28–30,34]. However, the results of our study are contrary to some studies. In this regard, Cantalapiedra-Hijar et al. [6] reported that DM intake was not impressed by increasing the dietary percentage of concentrate from 30% to 70% in goats. The results of Aguerre et al. indicated that a step-by-step increase in the dietary ratio of F/C (47:53, 54:46, 61:39, and 68:32) did not affect the DM intake of Holstein cows. Agle et al. [35] stated that there was no increase in DM consumption in dairy cows fed rations containing 52% and 72% concentrate. This issue can be because the animal consumes feed to supply its energy needs, and after supplying those needs, the appetite to eat decreases. Also, reducing the percentage of feed intake by the animal can also be effective in obtaining different results. Fodder constitutes half of the diet of ruminants, which affects the consumption of DM and, ultimately, the intake of energy by the animal. The contents of CP, NDF, and nonfiber carbohydrate (NFC) differ among different sources of forage, and because hay contains a higher amount of NFC, it is expected to provide more energy for the absorption of RDP in the rumen [18]. Thus, the efficiency of MPS when high-quality forage is consumed by the animal is often high (30–45 gm of MN per kg of digestible organic matter), while the efficiency of MPS with conventional low-quality forage has been recorded at 20 gm of MN per kg of digestible organic matter [20,36]. However, according to the results of Table 2, the decreased dietary ratio of fodder to concentrate in Afshari lambs increased feed consumption to compensate for the negative effects caused by the lack of alfalfa nutrients and to maintain physical satiety in livestock.

Measuring the urinary excretion of PD is a simple method to estimate the ruminal MPS [8,14,17,26]. The usual feeds of ruminants have a small amount of nucleic acids, which are almost completely decomposed inside the rumen under the influence of rumen microorganisms. Therefore, the nucleic acids introduced into the intestine are of ruminal microbial origin [11]. Microbial nucleic acids are degraded in the intestine after passing through the rumen. Purine nucleosides and free purines absorbed through the intestine will be decomposed into PDs (hypoxanthine, xanthine, uric acid, and allantoin) under the influence of the xanthine oxidase enzyme and finally excreted in the urine. PDs introduced into the bloodstream can also originate from the breakdown of tissue nucleic acids or an endogenous source [2]. This part of PDs that originates from tissues is called endogenous PDs, and a part of PDs that originates directly from nucleic acids is called exogenous PDs. Urinary nitrogen excretion is a factor in nitrogen absorption and recycling to the intestine, which in turn is related to the diet composition of the animals and ruminal feed efficiency [7,8,37].

According to the results of Table 3, a direct relationship between mean body weight and DOMI, DOMR, PD, and MN yield is observed. Also, the means of traits in diets 2 and 3 (the high level of concentrate) were higher than in diets 1 and 3 (the low level of concentrate). It is worth considering that, despite a linear relationship between the traits of body weight, DM intake, and MN production, the index of nitrogen yield per kg of DOMR does not necessarily follow this relationship. Therfore, despite the higher mean of MN yield as gN/d in diets 2 and 4 (10.75 and 11.48 *vs.* 9.51 and 11.32), the mean of MN yield as gN/kg DOMR in these diets was lower than in diets 1 and 3 (20.24 and 20.32 *vs*. 20.81 and 22.37) and almost equal. This issue implies the necessity of correcting the data based on the actual organic matter digestibility in the rumen [6,18,38]. The results of the current experiment indicated that increasing the amount of concentrate in the diet and removing alfalfa resulted in the elevated excretion of allantoin, uric acid, xanthine, hypoxanthine, and all of their derivatives, purine bases, in urine. So growing lambs that consumed a 90% concentrate diet, 10% wheat straw, and 0% alfalfa had the highest levels of allantoin, uric acid, xanthine, hypoxanthine, and total derivatives of purine bases with 9.25 mmol/day, 2.55 mmol/day, 1.45 gm/day, and 13.25 mmol/day in urine, respectively (). The increase in the amount of allantoin excreted simultaneously with the increase in concentrate, and dietary removal of alfalfa indicates an increase in the amount of ruminal digestible and fermentable organic matter. On the other hand, dietary increases in concentrate caused an increase in daily organic matter intake, which is a stimulus for increasing the amount of urinary excretion of allantoin [6,19,32]. As noted before, the efficiency of MPS when high-quality forage is consumed by the animal is often greater than when low-quality forage is used [20,36]. Coordinated with the findings of Carro et al. [8], the results of this study indicate that a decreased F/C ratio in the experimental diets increased feed intake to compensate for the negative effects of a lack of dietary high-quality forage and resulted in more urinary excretion of allantoin and total PD.

Ma et al. [14] investigated the relationship between MN yield and urinary excretion of PDs from the rumen in Dorper × thin-tailed Han crossbred sheep. They reported that the efficiency of MN synthesis (gN/kg organic matter apparently digested in the rumen) was not affected by feed intake, but urinary PD decreased significantly following decreasing feed intake (*p* < 0.05). Urinary PD excretion was linearly correlated with DOMI and MN yield (*r*^2^ = 0.94, *p* < 0.001). The results suggest that urinary PD excretion is an accurate indicator for ruminal MN synthesis, and the MN (gm/day) = 0.030 + 0.741 × PD (mmol/day) equation is proposed to predict the ruminal MN yield for Chinese sheep.

Ma et al. [17] also investigated the effects of various dietary F/C ratios on PD urinary excretion and ruminal MN yields in Dorper × thin-tailed Han crossbred sheep. Digestibility trials were conducted, and MN yields were estimated using either nitrogen or PD as markers. Urinary excretion of allantoin and total PD increased (*p* < 0.05) after decreasing the F/C ratio. Urinary excretion of uric acid or xanthine plus hypoxanthine is unaffected by the F/C ratio (*p* > 0.05). MN yields estimated using marked nitrogen were greater than those predicted from urinary PD (12.5 *vs.* 11.5 gm/day, *p *< 0.05), but the former was more variable than the latter (SE = 0.66 *vs.* 0.45, respectively). A linear correlation existed between MN estimated by marked nitrogen and urinary excretion of PD: MN (gm/day) = −0.521 + 1.493 PD (mmol/day) (*r*^2 ^= 0.86, *p *< 0.05). The ratio between PD nitrogen to urinary nitrogen as purine nitrogen index (PNI) was linearly correlated with nitrogen capture efficiency calculated from either marked N or PD (*r*^2 ^= 0.60 and 0.77, respectively). The results suggest that urinary PD is an accurate indicator for ruminal MN in Dorper crossbred sheep and that PNI reflects the conversion of nitrogen degradation to MN in the rumen.

According to the results presented in Table 4, the amount of allantoin, uric acid, xanthine, and hypoxanthine excretion separately, total PDs, absorbed microbial purines, MN, and synthesized microbial protein in growing lambs increased significantly due to increasing the ratio of concentrate in the diets (*p *< 0.01). In all research, allantoin is the most important product of purine catabolism and the main derivative excreted in the urine, so it is well possible to estimate the amount of microbial protein entered into the small intestine [4,6,8,11,25,26,32,37]. In this regard, Dipu et al. [37] measured the ruminal MPS in the buffalo using the PD excretion index, and with a dietary ratio of 40% wheat straw and 60% concentrate, all experimental animals were fed at levels of 95%, 80%, 60%, and 40% of the daily optional intake. The results showed a positive relationship between the urinary excretion of PD and feed consumption by animals.

According to our results, the total level of absorbed microbial purines, MN, and ruminal MPS were significantly affected by an increased dietary ratio of concentrate (*p *< 0.01). As expected, growing lambs fed diet 4 (90% concentrate, 10% wheat straw, and 0% alfalfa) had significantly the highest level of MPS (71.80 gm/day) at 45 days of age and the highest amount of absorbed microbial purines (15.78 mmol/day) (Table 4). In fact, duodenal purine bases as a microbial marker are effectively absorbed in the small intestine, and most urinary metabolites are excreted in the urine, with different urinary reabsorption efficiency among animal species [25,26].

Most of the feed nitrogen is converted into microbial protein through ruminal fermentation; following the increase in concentrate, it is absorbed in the intestine along with the feed protein and leads to improved performance. Low rumen pH can be problematic for ruminant animals fed high concentrate or grain diets, especially when the acid produced exceeds the buffering capacity of the rumen [19,30,34,39]. However, in this research, the complete replacement of straw with alfalfa has prevented the premature reduction of rumen pH. Also, increasing the level of concentrate and subsequently increasing the DM consumed by the animal led to higher MPS in diet 4 compared to other experimental diets. Previous studies suggest increasing dietary protein and energy can increase nitrogen concentration and ruminal MPS [3,6,8,14]. Several studies have used PD urinary excretion to estimate the MN outflow into the intestine, with a strong correlation [10,12,17]. Timmermans et al. [10] found that the outflow of MN to the intestine was not significantly related to urinary allantoin excretion, but for uric acid excretion, the relationship was significant. A strong positive linear relationship between the outflow of MN into the duodenum and the total excretion of PDs has been documented [7,8,26]. Because MN was estimated using the number of purine bases in the digestive material in the duodenum, it was determined that there is a positive correlation between MN and the PD excretion in the urine of animals [13,37,40]. The results of this study confirm the previous observations, so a significant relationship between total PDs and MPS was observed. Furthermore, according to our results, there was a strong relationship between daily feed intake of digestible organic matter and MN production, as well as total urinary PD excretion.

## Conclusion

Currently, due to environmental problems, most countries are facing a shortage of fodder. In this situation, it is very important to strictly follow the concentrate ratio as well as the essential points related to the F/C ratio. The results of the present study indicated that, by increasing the concentrate ratio in the experimental diets, urinary excretion of PDs and ruminal microbial protein synthesized increased, and accordingly, the performance of growing lambs, including daily gain, feed intake, DOMI, fermentable DOM, and microbial N yield, improved. Therefore, monitoring PD urinary excretion is an accurate and acceptable indicator for MPS estimation and improving the performance, digestibility, and feed productivity of fattening lambs.

## List of abbreviations

gm, gram; kg, kilogram; mmol, millimole; NS, not significant; *p*-value, probabilty value; SEM, standard error of mean; SE, standard error; vs*.*, versus.
